# Low levels of serum ferritin and moderate transferrin saturation lead to adequate hemoglobin levels in hemodialysis patients, retrospective observational study

**DOI:** 10.1371/journal.pone.0179608

**Published:** 2017-06-29

**Authors:** Chie Ogawa, Ken Tsuchiya, Naohisa Tomosugi, Fumiyoshi Kanda, Kunimi Maeda, Teiryo Maeda

**Affiliations:** 1 Maeda Institute of Renal Research, Kawasaki, Kanagawa, Japan; 2 Biomarker Society, INC, Kawasaki, Kanagawa, Japan; 3 Department of Blood Purification, Tokyo Women’s Medical University, Tokyo, Japan; 4 Division of Systems Bioscience for Drug Discovery, Project Research Center, Medical Research Institute, Kanazawa Medical University, Ishikawa, Japan; Pennsylvania State University College of Medicine, UNITED STATES

## Abstract

**Background:**

Optimal iron levels in patients on hemodialysis are currently unknown, and a higher level than that for the healthy population is usually set for such patients considering the use of erythropoiesis-stimulating agents or the occurrence of chronic inflammation. However, excessive iron causes oxidative stress and impairment of its utilization by cells. Therefore we investigated the relationship between hemoglobin (Hb) level and iron status in hemodialysis patients to identify the optimal iron levels for patients undergoing hemodialysis.

**Methods:**

A total of 208 outpatients on maintenance hemodialysis were followed up between July 2006 and June 2007. Men accounted for 64.9% cases [mean age, 59.3 ± 13.1 years and median dialysis history, 7.7 (3.6–13.2) years], and diabetic nephropathy accounted for 25.0% cases. Hemoglobin level was measured twice a month and serum ferritin, serum iron, and total iron-binding capacity were measured once a month. The doses of recombinant human erythropoietin and low-dose iron supplement were adjusted to maintain a hemoglobin level of 10–11 g/dL, according to the guidelines of the Japanese Society for Dialysis Therapy. Hepcidin was measured at baseline. Using the mean values for 1-year period, the relationships among hemoglobin, serum ferritin levels, and transferrin saturation levels were investigated based on a receiver operating characteristic curve and a logistic regression model. In addition, the correlations among serum ferritin, transferrin saturation, and hepcidin levels were analyzed by Pearson product—moment correlation coefficient and linear regression model.

**Results:**

By receiver operating characteristic curve, the cutoff point of serum ferritin and transferrin saturation levels with a hemoglobin ≥10 g/dL showed <90 ng/mL (sensitivity: 69.1%, specificity: 72.1%, p < 0.001) and ≥20% (sensitivity: 77.6%, specificity: 48.8%, p = 0.302).

Upon logistic regression model analysis with a hemoglobin ≥10 g/dL as the endpoint, the analysis of odds ratios relative to a group with serum ferritin ≥90 ng/mL and transferrin saturation <20% revealed that the group with serum ferritin <90 ng/mL and transferrin saturation ≥20% had the highest ratio: 46.75 (95% confidence interval: 10.89–200.70, p < 0.001). In Pearson product—moment correlation coefficient, hepcidin showed a strong positive correlation with serum ferritin [r = 0.78 (95% confidence interval: 0.72–0.83, p < 0.001)] and a weak positive correlation with transferrin saturation [r = 0.18 (95% confidence interval: 0.04–0.31, p = 0.010)]. In the multivariable analyses of the linear regression model, a positive relationship was shown between hepcidin and serum ferritin [β-coefficient of 0.30 (95% confidence interval: 0.27–0.34, p < 0.001)]; however, no relationship was shown with transferrin saturation [β-coefficient of 0.09 (95% confidence interval: −0.31–0.49, p = 0.660)].

**Conclusions:**

In this study, the iron status of serum ferritin <90 ng/mL and transferrin saturation ≥20% was optimal in hemodialysis patients receiving recombinant human erythropoietin for anemia therapy. This result indicates that the threshold values for the optimal iron status may be lower than those currently recommended in iron-level management guideline.

## Introduction

Optimal iron levels in patients on hemodialysis (HD) remain unclear. In particular, there are large differences in iron-level management implemented during anemia therapy for HD patients between western countries and Japan. The data of Dialysis Outcomes and Practice Patterns Study (DOPPS) showed the mean serum ferritin (s-ft) and Hb to be 600–700 ng/mL and 11.0–11.4 g/dL, respectively, in western countries in 2011 [1]. Conversely, according to a statistical study of the Japan Society for Dialysis Therapy conducted at the end of 2012, a mean s-ft of 144.31 ± 261.20 ng/mL, with s-ft < 100 ng/mL accounting for 58.4% of the study population, and Hb of 10.60 ± 1.28 g/dL were recorded [2]. The target Hb level presented by the Japan Society for Dialysis Therapy in 2008 was 10.0 g/dL–11.0 g/dL [3] The blood sampling is performed in the supine position on the first HD session of the week in Japan, whereas it is performed in the sitting position on the second HD session of the week in western countries. According to the study in Japan, Hb levels on the first HD session of the week were lower than those on the second HD session of the week, and Hb levels in the supine position were 94.4% of that in the sitting position [3]. Considering these facts, we do not believe that anemia therapy in Japan is inferior to that in western countries. It appears that Hb is able to be controlled at lower body iron levels in Japan than those in western countries.

Iron overload leads to oxidative stress, which may result in arteriosclerosis [4–6], opportunistic infection [7], and carcinogenesis [8]. High s-ft groups are reported to show a high level of oxidative stress in HD patients [6, 9, 10]. Anraku et al. reported that intravenous iron administration using 40 mg of ferric saccharate every HD session increased advanced oxidation protein product levels [9]. Maruyama et al indicated that the same treatment increased 8-OHdG levels [10]. Thus, these studies suggested that even common intravenous iron treatment might induce oxidative stress. However, it was shown by review of Kidney Disease Improving Global Outcomes that intravenous iron administration of doses of up to 400 mg/month had lower death rates compared to doses >400 mg/month [11]. We believe that more research is needed regarding this issue.

The intake and output of iron per day is only 1–2 mg corresponding to 3–4 g of iron content within the body. Iron metabolism takes place in an almost enclosed cycle. The annual amount of iron loss in HD patients owing to HD or blood drawing is considered to be between 1 and 2 g [12, 13]. Thus, the administration of iron dose equivalent to its loss is necessary. However, a recent study [14] reported the annual iron loss owing to HD or blood sampling decreased to approximately 500 mg with recent development of dialysis therapy. This suggests that the required dose of iron replacement as well as stored iron may be less than that previously assumed. It has also been revealed that excessive iron intake leads to a reduction in the efficiency of its use [15]. These updates have made us consider the need for a review of the optimal iron content in HD patients to avoid oxidative stress owing to excessive iron intake and achieve effective iron use. Therefore, we investigated the correlation between Hb level and iron status.

## Materials and methods

### Patients

Study subjects comprised a total of 208 patients who were receiving maintenance HD as outpatients at the Maeda Institute of Renal Research (Kanagawa, Japan) between July 2006 and June 2007. Patients received HD three times a week for 4–5 h each in all the cases.

All patients provided informed consent permitting data sampling and analysis at the time of initiation of the dialysis therapy. The protocol for the study was approved by the ethics committee of the Biomarker Society, INC, comprising 7 committees, including outside experts.

### Methods

Blood sampling was performed at the beginning of each week when initiating HD. Anemia-related data were measured twice per month and s-ft, serum iron (Fe), total iron-binding capacity (TIBC), serum albumin(s-Alb), and C-reactive protein (CRP) were measured once per month. Transferrin saturation (TSAT) was calculated on the basis of iron and TIBC (TSAT = Fe/TIBC × 100). In addition, Kt/V was measured by a single pool calculation once a month. S-Alb was measured by the BCG method.

With a target Hb level set at 10–11 g/dL in accordance with the guidelines of the Japan Society for Dialysis Therapy, recombinant human erythropoietin (rHuEPO) and iron preparation were administered as the anemia therapy, and s-ft < 30 mg/dL was used as the criterion to initiate iron administration. The administered iron supplement was 40 mg of ferric saccharate once a week for 2–6 weeks.

The mean values of the respective data for a period from July 2006 to June 2007 were calculated and a relationship between Hb level and iron status was examined.

At study initiation, hepcidin levels along with Hb levels and iron-related measurements were made. Hepcidin was measured using the quantitative method of liquid chromatography coupled with tandem mass spectrometry (LC-MS/MS) [16].

### Statistical analysis

Analyses were performed with the SAS system software, version 9.4 (SAS Institute, Cary, N.C., USA). Data were summarized as the mean ± standard deviation (SD), medians with interquartile ranges and frequency. To clarify the optimal cut points for ferritin and TSAT regarding Hb≥10g/dl, receiver operating characteristic (ROC) analysis with Youden index was applied. To evaluate the impact of the ferritin and TSAT on Hb≥10g/dl, we applied the univariable and multivariable logistic regression model. To assess the relationship for ferritin and TSAT with hepcidin, the Pearson product—moment correlation coefficient was used. Furthermore, univariable and multivariable linear regression model were also applied. The multicollinearity in the multivariable model was examined using the regression diagnostic analysis. Two-tailed p values of <0.05 were considered to indicate a statistically significant difference. All analyses were performed at an independent biostatistics and data center (STATZ Institute, Inc., Tokyo, Japan).

## Results

### Patients

Table 1 showed the basic characteristics. The mean age at the start of the study was 59.3 ± 13.1 years. A total of 135 patients were male and 73 patients were female. The median duration of dialysis was 7.7 (3.6–13.2) years. The primary disease was chronic glomerulonephritis in 117 patients (56.3%), diabetic nephropathy in 52 patients (25.0%), nephrosclerosis in 16 patients (2.7%), polycystic kidney disease in seven patients (3.4%), rapidly progressive glomerulonephritis in three patients (1.4%) and others in 13 patients (6.3%). The mean Hb, corpuscular volume, corpuscular hemoglobin, and reticulocytes were 10.3 ± 0.9 g/dL, 94.6 ± 5.8 fL, 31.1 ± 2.1 pg, and 16.7% ± 8.4%, respectively. The median s-ft was 50.6 (22.7–125.0) ng/mL, and the mean values of Fe, TIBC, and TSAT were 59.4 ± 19.6 μg/dL, 243.3 ± 43.0 μg/dL, and 24.7± 9.4%, respectively. The median Hep 25 was 29.8 (9.5–56.9) ng/mL. The mean values of urea nitrogen, creatinine, albumin, calcium, and phosphorous were 69.7 ± 13.2 mg/dL, 12.3 ± 2.6 mg/dL, 3.9 ± 0.3 g/dL, 9.4 ± 0.8 mg/dL, and 5.8 ± 1.2 mg/dL, respectively. The median CRP was 0.06 (0.03–0.21) mg/dL. The mean rHuEPO was 3909 ± 2725 IU.

**Table 1:** Patient characteristics.

variables	value
	(n = 208)
Age(years)	58.9±12.9
Gender	
Men	135
Women	73
Duration of dialysis (years)*	7.88 (3.7–14.4)
Primary diagnosis	
Chronic glomerulonephritis	117
Diabetes nephropathy	52
Renal sclerosis	16
Polycystic Kidney	7
RPGN	3
Other	13
Kt/V	1.32±0.22
Hemoglobin (g/dL)	10.3±0.9
MCV (fL)	94.6±5.8
MCH (pg)	31.1±2.1
Reticlocyte (%)	16.7±8.4
serum-Ferritin (ng/mL)*	50.6 (22.7–125)
Iron (μg/dL)	59.4±19.6
TIBC (μg/dL)	243.3±43.0
Transferrin saturation (%)	24.7±9.4
Hepcidin (ng/mL)*	29.8 (9.5–56.9)
Urea nitrogen(mg/dL)	69.7±13.2
Creatinin(mg/dL)	12.3±2.6
Albumin(g/dL)	3.9±0.3
Calcium(mg/dL)	9.4±0.8
Phosphoric(mg/dL)	5.8±1.2
C-reactive protein (mg/dL)*	0.06 (0.03–0.20)
rHuEPO (IU/week)	3909±2725

Mean ± SD and median and interquartile range (IQR)*

Abbreviations: RPGN, rapidly progressive glomerulonephritis; MCV, mean corpuscular volume; MCH, mean corpuscular hemoglobin; TIBC, total iron-binding capacity; rHuEPO, recombinant human erythropoietin.

### Examination of Hb control and iron status

**Fig 1:**
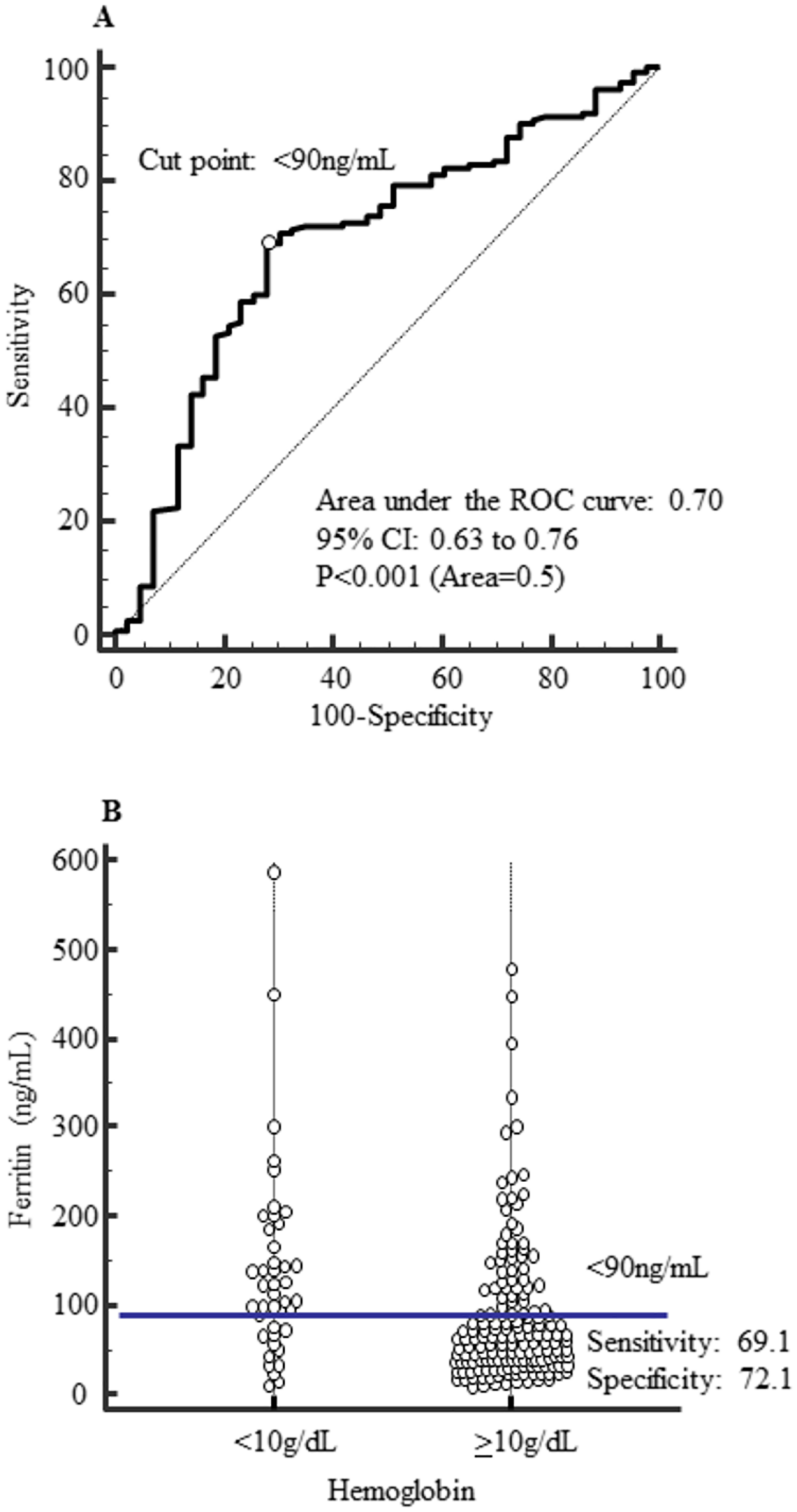
Diagnostic accuracy of serum ferritin. (A) The ROC curve of serum ferritin with Hemoglobin ≥10g/dL. (B) Dot plot of serum ferritin in Hemoglobin <10g/dL and ≥10g/dL. The cutoff point for s-ft was found to be below 90 ng/mL (sensitivity: 69.1%, specificity: 72.1%, p < 0.001).

**Fig 2:**
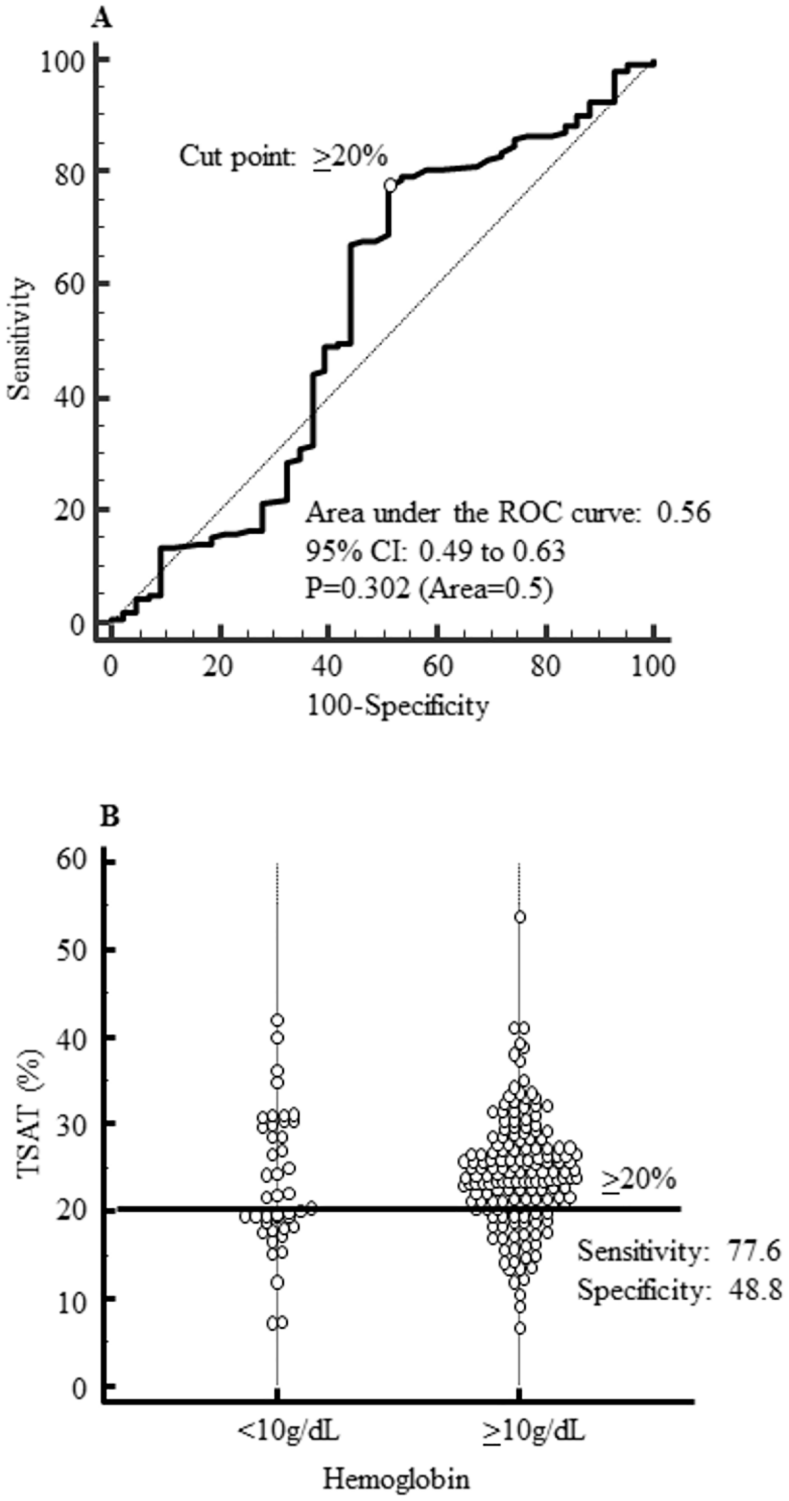
Diagnostic accuracy of transferrin saturation. (A) The ROC curve of transferrin saturation with Hemoglobin ≥10g/dL. (B) Dot plot of transferrin saturation in Hemoglobin <10g/dL and ≥10g/dL. The cutoff point for TSAT was found to be at least 20% (sensitivity: 77.6%, specificity: 48.8%, p = 0.302).

Upon ROC analysis with a Hb level of at least 10 g/dL set as an endpoint, the cutoff point for s-ft was found to be below 90 ng/mL (sensitivity: 69.1%, specificity: 72.1%, p < 0.001) (Fig 1), whereas the cutoff point for TSAT was found to be at least 20% (sensitivity: 77.6%, specificity: 48.8%, p = 0.302) (Fig 2).

Logistic model analysis with a Hb level of at least 10 g/dL set as an endpoint showed that the odds ratio of the group with s-ft < 90 ng/mL to the group with s-ft ≥ 90 ng/mL was significantly high: 5.31 [95% confidence interval (CI): 2.59–11.02, p < 0.001] in univariable analysis and 8.13 (95% CI: 3.49–18.90, p < 0.001) in multivariable analysis. The odds ratio of the group with TSAT ≥ 20% to the group with TSAT < 20% was significantly high: 3.05 (95% CI 1.50–6.21, p = 0.002) in univariable analysis and 5.46 (95% CI: 2.30–12.95, p < 0.001) in univariable analysis. Odds ratios were obtained by analysis relative to the group with s-ft ≥ 90 ng/mL and TSAT < 20%. The ratio for the group with s-f ≥ 90 ng/mL and TSAT ≥ 20% was 6.66 (95% CI: 1.88–23.54, p = 0.003). The ratio for the group with s-ft < 90 ng/mL and TSAT < 20% was 10.31 (95% CI: 2.58–41.19, p < 0.001). The ratio for the group with s-ft < 90 ng/mL and TSAT ≥ 20% was highest: 46.75 (95% CI: 10.89–200.70, p < 0.001) (Table 2).

**Table 2:** Logistic model analysis with a Hb≥10 g/dL set as an endpoint.

Variables	No. of Patients	No. of Hb≥10g/dL (%)	Univariable analysis			Multivariable analysis		
			Unadjusted Odds ratio	95% CI	P-value	Adjusted Odds ratio	95% CI	P-value
**Ferritin(ng/mL)**								
≥**90**	80	50 (62.5%)	1.00					
**<90**	128	115 (89.8%)	5.31	(2.56–11.02)	<.001	8.13	(3.49–18.90)	<.001
**TSAT(%)**								
**<20**	53	34 (64.2%)	1.00					
**≥20**	155	131 (84.5%)	3.05	(1.5–6.21)	0.002	5.46	(2.3–12.95)	<.001
**Ferritin(ng/mL), TSAT(%)**								
≥**90**, <**20**	15	4 (26.7%)	1.00					
≥**90**, ≥**20**	65	46 (70.8%)	6.66	(1.88–23.54)	0.003	-	-	-
<**90**, <**20**	38	30 (78.9%)	10.31	(2.58–41.19)	<.001	-	-	-
<**90**, ≥**20**	90	85 (94.4%)	46.75	(10.89–200.70)	<.001	-	-	-

**Fig 3:**
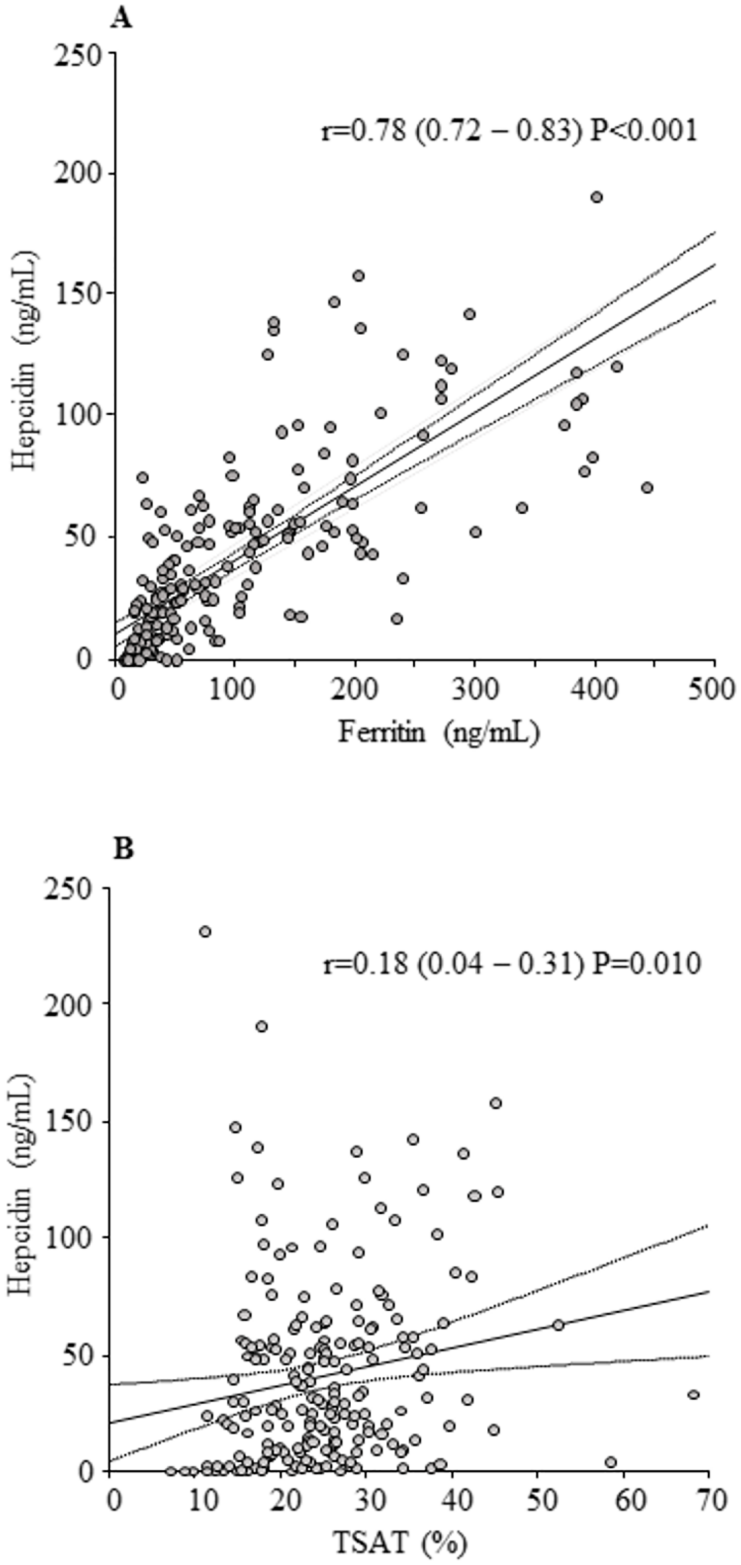
The relationship for serum ferritin and transferrin saturation with hepcidin. (A) Serum ferritin showed a strong positive correlation with hepcidin [r = 0.78 (95% CI: 0.72–0.83, p < 0.001)]. (B) Transferrin saturation indicated a weak positive correlation with hepcidin [r = 0.18 (95% CI: 0.04–0.31, p = 0.010)].

Although hepcidin showed a strong positive correlation with s-ft [r = 0.78 (95% CI: 0.72–0.83, p < 0.001)], it only showed a weak positive correlation with TSAT [r = 0.18 (95% CI: 0.04–0.31, p = 0.010)] (Fig 3). In a linear regression model, a positive relationship between hepcidin and s-ft was also observed in both univariable and multivariable analyses corrected for TSAT [β-coefficients of 0.30 (95% CI: 0.27–0.34, p < 0.001) and 0.30 (95% CI: 0.27–0.34, p < 0.001)]. However, although TSAT showed a positive relationship in univariable analysis [β-coefficients of 0.80 (95% CI: 0.19–1.41, p = 0.010)], it did not show such a relationship in multivariable analyses corrected for s-ft [β-coefficients of 0.09 (95% CI: –0.31–0.49, p = 0.660)] (Table 3).

**Table 3:** The relationship for ferritin and TSAT with hepcidin by multiple linear regression model.

Independent variables	No. of Patients	Univariable analysis			Multivariable analysis		
		Unadjusted Regression Coefficient	95% CI	P-value	Adjusted Regression Coefficient	95% CI	P-value
**Ferritin (per 1ng/mL increase)**	204	0.30	(0.27 to 0.34)	<.001	0.30	(0.27 to 0.34)	<.001
**TSAT (per 1% increase)**	204	0.80	(0.19 to 1.41)	0.010	0.09	(-0.31 to 0.49)	0.660

## Discussion

We conducted an analysis using Hb ≥10.0 g/dL as the endpoint for setting the target Hb level for anemia therapy at 10.0–11.0 g/dL according to Japanese guidelines. As a result, the cutoff points in ROC analysis were s-ft < 90 ng/mL and TSAT ≥ 20%. With an endpoint set at Hb ≥10.0 g/dL in the logistic regression model as well, the group with s-ft <90ng/mL had a significantly higher odds ratio of 8.13 than the group with s-ft ≥90 ng/mL. Likewise, the group with TSAT ≥20% showed a significantly higher odds ratio of 5.46 than the group with TSAT <20%. Thus, these results showed that the probability to achieve Hb ≥10.0 g/dL was significantly higher in the group with s-ft <90ng/mL or TSAT ≥20% in HD patients. Comparing the analysis of s-ft and TSAT, the odds ratio of the group with s-ft <90ng/mL was higher than that of the group with TSAT ≥20% (8.13 versus 5.46). Moreover, in ROC analysis, area under the curve, sensitivity and selectivity of s-ft was higher compared with the corresponding values of TSAT. Therefore s-ft levels might influence more on the control of Hb levels in comparison with that of TSAT. In analysis with s-ft and TSAT combined, s-ft ≥90 ng/mL and TSAT <20% were set as the reference group. The odds ratio using Hb ≥10.0 g/dL as the endpoint showed that the group with s-ft <90 ng/mL and TSAT ≥20% was highest, with >90% cases in the group achieving Hb ≥10.0 g/dL. The odds ratio of the analysis that combined s-ft and TSAT was higher than that of the analysis with s-ft alone. It was thought that the management of iron status using a combination of s-ft with TSAT was useful for the control of Hb. This study indicated the iron status of s-ft ≥90 ng/mL and TSAT <20% was good for treating anemia in HD patients.

Furthermore, analyses of the relationships between s-ft or TSAT and hepcidin were performed, in which a strong positive correlation between s-ft and hepcidin was confirmed.

Hepcidin, which was discovered in 2000, is a peptide that controls the body iron content and metabolism.

Iron plays critical roles, such as oxygen transport and energy production, in the body and it is an essential element in living organisms. At the same time, non-protein-bound iron also called free iron has high toxicity and causes oxidative stress. In line with this, the strict control mechanism of free iron levels is seen in the body. A total of 20–25 mg of iron is released in the circulation every day. The majority of the released iron is provided by reticuloendothelial macrophages as part the metabolism of hemoglobin from red blood cells, with only 1–2 mg of iron being released into the circulation per day through intestinal iron absorption. Hepcidin regulates body iron metabolism by degrading ferroportin, which is involved in cellular iron efflux and intestinal iron absorption. When hepcidin degrades ferroportin, it causes the cessation of iron efflux from cells into the blood, resulting in a reduction in iron use [17]. This means that excessive levels of hepcidin can lead to impairment of the efficiency of iron use, leading to deficiency of Fe, which is required for hematopoiesis.

Hepcidin has three peptide types, hepcidin-20, -22 and -25. Hepcidin-25 plays roles in iron metabolism mainly. However, these peptide types are difficult to distinguish by antibody assays. Therefore, we measured hepcidin using LC-MS/MS, which only allowed for the quantification of hepcidin-25.

Currently, iron status in the body is generally estimated according to s-ft and TSAT. S-ft is ft released partially from the cell into the blood. Although s-ft is a good indicator of the iron stores in the body, in the presence of inflammation, s-ft levels shows an elevated value [18]. On the other hand, although TSAT represents available serum iron levels in the body, it shows diurnal variation and is affected by the nutrition status [19] and/or the presence of inflammation [20]. Accordingly, relying on either indicator alone might not lead to accurate results regarding the body iron status, which explains why s-ft and TSAT are currently combined as parameters to estimate the body iron status. Thus, we investigated both s-ft and TSAT to study the optimal iron content when rHuEPO is used in anemia therapy.

The threshold indicative of absolute iron deficiency in healthy individuals is regarded to be s-ft < 12 ng/mL [21]. Meanwhile, the lower threshold for iron management in HD patients is set at s-ft 200 ng/mL [22] according to the Kidney Disease Outcome Quality Initiative (KDOQI) and s-ft < 100 ng/mL and TSAT < 20% [23] according to the European Best Practice Guidelines. Thus, the thresholds for HD patients are set higher than those for healthy individuals. Additionally, intravenous infusion has been recommended for iron administration as it is considered to increase Hb more than orally-consumed iron [23, 24]. However, it has been recently revealed that even in cases with anemia of chronic disease, when the condition is complicated by iron deficiency anemia, the hepcidin level is significantly lower than that in cases with anemia of chronic disease alone, showing that iron absorption from recirculation of macrophages and intestinal iron absorption is possible [25]. A study [26] of intestinal iron absorption in HD patients using isotopes has reported that 20% or more of degree of iron absorption was observed at s-ft < 30 ng/mL. In the same study, however, the iron absorption was decreased to 2.52% at s-ft 96 ng/mL. This suggests the possibility that body iron content is sufficient at s-ft ≥ approximately 100 ng/mL, leading to a reduction in iron absorption. According to Nakanishi et al. [27], recirculation of iron from macrophages was decreased at s-ft 50 ng/mL and hepcidin 11.3 ng/mL or higher levels. In the same study, at s-ft 100 ng/mL and hepcidin 18.6 g/mL, recirculation of iron from macrophages was 20 mg/day and intestinal iron absorption was 1 mg/day, being the lower limit of normal. These data also indicate that the efficiency of iron use decreases at s-ft > 100 ng/mL. Moreover, there are reports that erythropoietin response is poor at a high level of hepcidin [28] and that a negative correlation between hepcidin and reticulocytes was observed in HD patients [29]. These reports suggest that excessive hepcidin might lead to an unfavorable situation in dialysis patients being treated for anemia.

According to a study [30] in which s-ft and hepcidin were measured in healthy individuals and HD patients, strong positive correlations between s-ft and hepcidin were observed in both groups and the s-ft/ hepcidin values of both groups were similar. Another previous study reported that because interluekin-6 induces hepcidin [31], hepcidin level is more likely to increase in dialysis patients with chronic inflammation than in healthy individuals [16]. This implies that in dialysis cases in which inflammation has been successfully controlled, the hepcidin regulates body iron content with a capacity comparable to that of healthy individuals. In the same study, hepcidin < 25 ng/mL was confirmed in 75% healthy individuals and 50% HD patients.

Another study reports that when iron was orally administered to patients diagnosed with iron deficiency, the response to the treatment was poor in the cases of s-ft > 30 ng/mL and hepcidin > 20 ng/mL [32]. Furthermore, anemia correction effects for oral iron administration were poor in the groups with mean values of s-ft and hepcidin in the range of 30 ng/mL on HD patients [33]. These studies indicate that s-ft does not need to be maintained at a level as high level as that previously considered and that iron deficiency affects hematopoiesis only mildly if s-ft > 30 ng/mL and hepcidin > 20–30 ng/mL are maintained. It is also noted that in these studies, hepcidin was measured using LC-MS/MS, as used by us in the present study.

A strong positive correlation between s-ft and hepcidin was observed in our study as well, in which the hepcidin level of 25–30 ng/mL corresponded to s-ft of approximately 50–90 ng/mL.

According to the studies on both intestinal iron absorption and the reuse of iron from macrophages and hepcidin, s-ft < 90 ng/mL is considered to be an appropriate value for smooth iron supply into the blood.

TSAT < 20% has long been used as an indicator of iron deficiency [34–36] and might suggest a state in which the iron content available for hematopoiesis is decreased. The Hb level was significantly decreased in cases with TSAT < 20% according to Japan-DOPPS (1999–2006) [37]. These data justify continued adherence to the existing data stating that maintaining TSAT ≥ 20% is recommended to maintain Hb ≥ 10 g/dL.

Although beyond the scope of this paper, it is noted that there are studies reporting oxidative stress involving ephemeralization of RBC survival [38, 39] or erythropoietin resistance [40]. Another study has reported that a high level of oxidative stress was observed in a group with s-ft ≥ 100 ng/mL [10]. Taken together, these studies imply that s-ft < 100 ng/mL might be a more appropriate level for preventing oxidative stress from affecting anemia.

In terms of data from large-scale studies, results from Japan-DOPPS (1999–2006) showed that the mean Hb was the highest at s-ft 0–49 ng/mL and that the mean Hb decreased as s-ft increased [37]. The analysis of iron status and Hb using the data of >140,000 patients held by the Japan Society for Dialysis Therapy showed that the Hb level was high at s-ft < 100 ng/mL and TSAT ≥ 20% [41]. This result was mostly consistent with our data.

In this study, however, there are some limitations. First, the study was a retrospective and observational study and the sample size was limited. These limitations might hinder a thorough consideration of confounding factors that can affect Hb levels. Next, although the target Hb level was set, anemia therapy depended on each doctor’s discretion. Given these limitations, we expect to conduct a large-scale prospective study on the effects of iron status on Hb levels for HD patients in the future.

In this study, the group with s-ft < 90 ng/mL and TSAT ≥ 20% showed the best iron status when rHuEPO was used for anemia therapy in HD patients. Because these levels are close to the threshold indicative of iron deficiency, we believe that careful iron management is necessary.

## Supporting information

S1 Fig Diagnostic accuracy of serum ferritin. (A) The ROC curve of serum ferritin with Hemoglobin ≥10g/dL. (B) Dot plot of serum ferritin in Hemoglobin <10g/dL and ≥10g/dL. The cutoff point for s-ft was found to be below 90 ng/mL (sensitivity: 69.1%, specificity: 72.1%, p < 0.001).
(TIF)

S2 Fig Diagnostic accuracy of transferrin saturation. (A) The ROC curve of transferrin saturation with Hemoglobin ≥10g/dL. (B) Dot plot of transferrin saturation in Hemoglobin <10g/dL and ≥10g/dL. The cutoff point for TSAT was found to be at least 20% (sensitivity: 77.6%, specificity: 48.8%, p = 0.302).
(TIF)

S3 Fig The relationship for serum ferritin and transferrin saturation with hepcidin. (A) Serum ferritin showed a strong positive correlation with hepcidin [r = 0.78 (95% CI: 0.72–0.83, p < 0.001)]. (B) Transferrin saturation indicated a weak positive correlation with hepcidin [r = 0.18 (95% CI: 0.04–0.31, p = 0.010)].
(TIF)

S1 Table Patient characteristics. (TIF)

S2 Table Logistic model analysis with a Hb>10 g/dL set as an endpoint. (TIF)

S3 Table The relationship for ferritin and TSAT with hepcidin by multiple linear regression model. (TIF)
