# Supplementary figures and images for: Low levels of serum ferritin and moderate transferrin saturation lead to adequate hemoglobin levels in hemodialysis patients, retrospective observational study
Source: PLoS One. 2017 Jun 29;12(6):e0179608. doi: 10.1371/journal.pone.0179608 (PMC5491034; doi:10.1371/journal.pone.0179608)

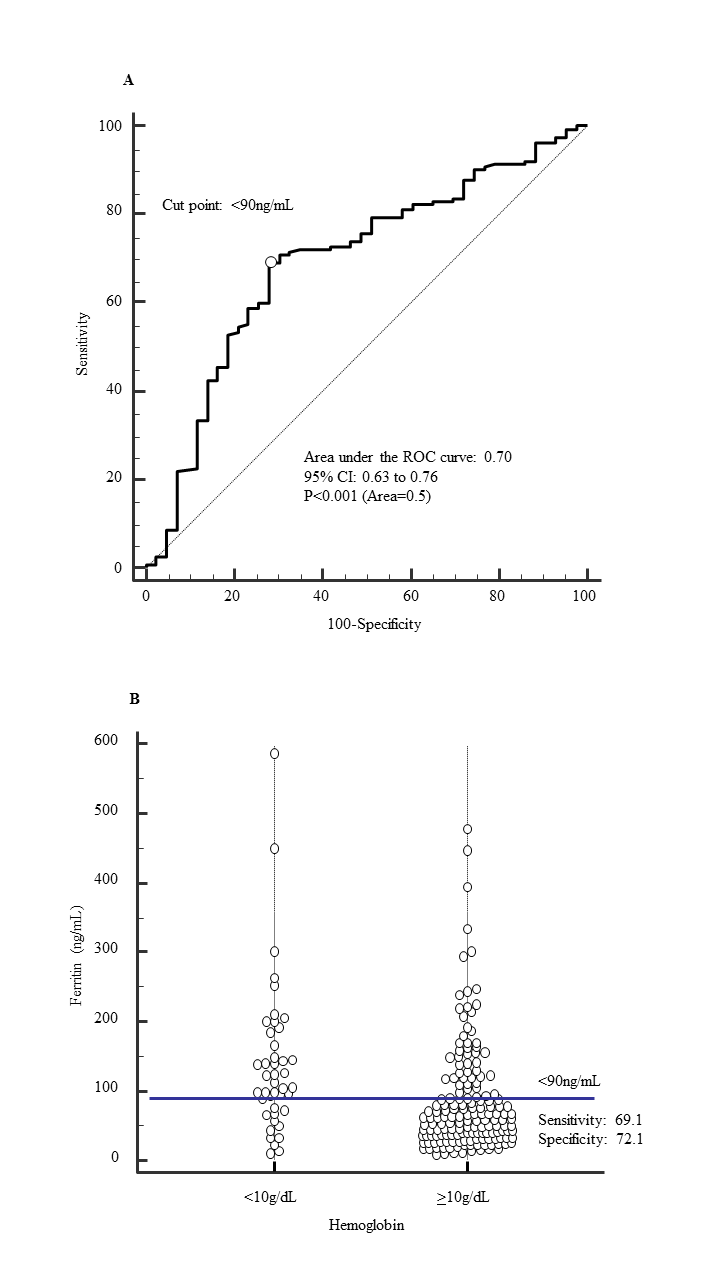

Supplement: S1 Fig — (A) The ROC curve of serum ferritin with Hemoglobin ≥10g/dL. (B) Dot plot of serum ferritin in Hemoglobin <10g/dL and ≥10g/dL. The cutoff point for s-ft was found to be below 90 ng/mL (sensitivity: 69.1%, specificity: 72.1%, p < 0.001). (TIF) [file pone.0179608.s001.tif]

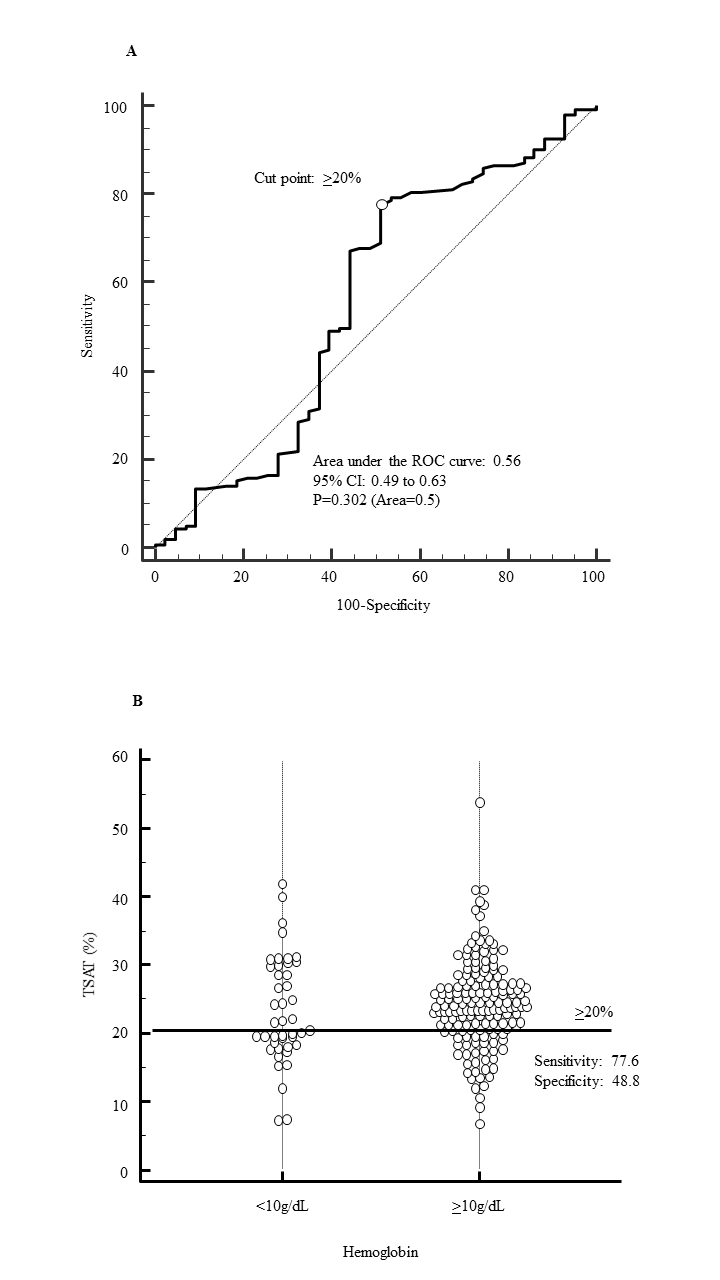

Supplement: S2 Fig — (A) The ROC curve of transferrin saturation with Hemoglobin ≥10g/dL. (B) Dot plot of transferrin saturation in Hemoglobin <10g/dL and ≥10g/dL. The cutoff point for TSAT was found to be at least 20% (sensitivity: 77.6%, specificity: 48.8%, p = 0.302). (TIF) [file pone.0179608.s002.tif]

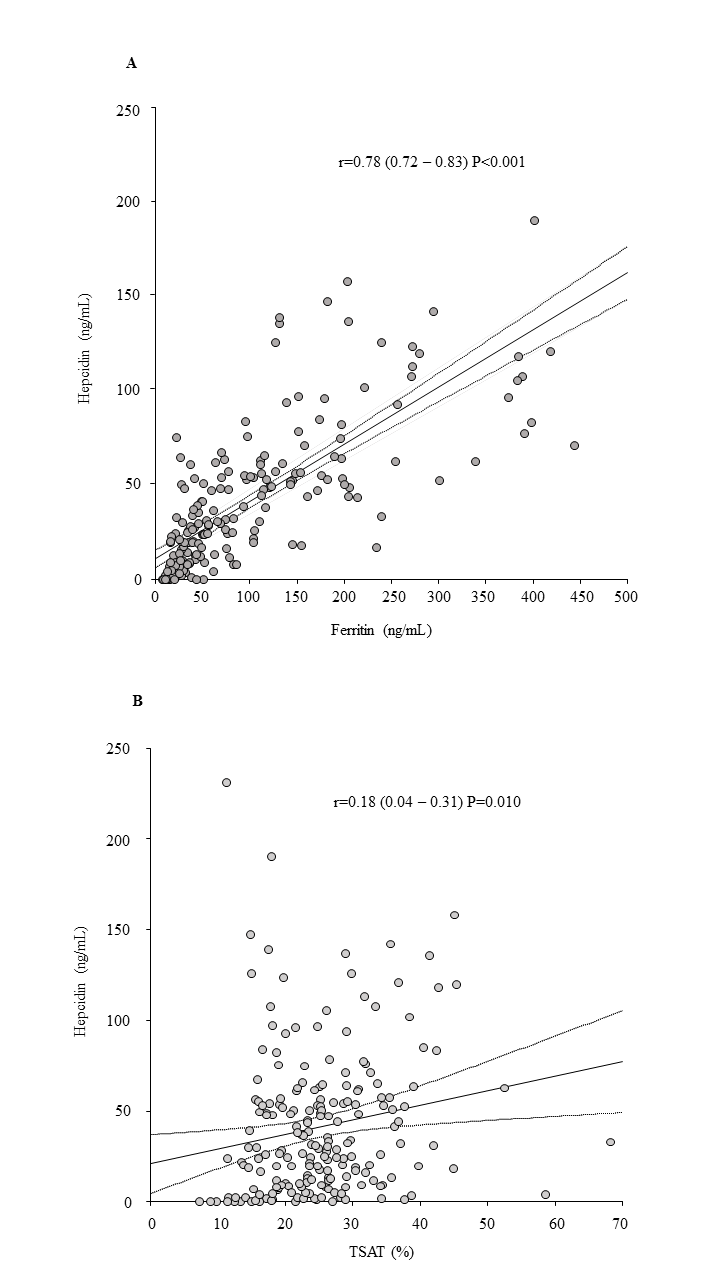

Supplement: S3 Fig — (A) Serum ferritin showed a strong positive correlation with hepcidin [r = 0.78 (95% CI: 0.72–0.83, p < 0.001)]. (B) Transferrin saturation indicated a weak positive correlation with hepcidin [r = 0.18 (95% CI: 0.04–0.31, p = 0.010)]. (TIF) [file pone.0179608.s003.tif]

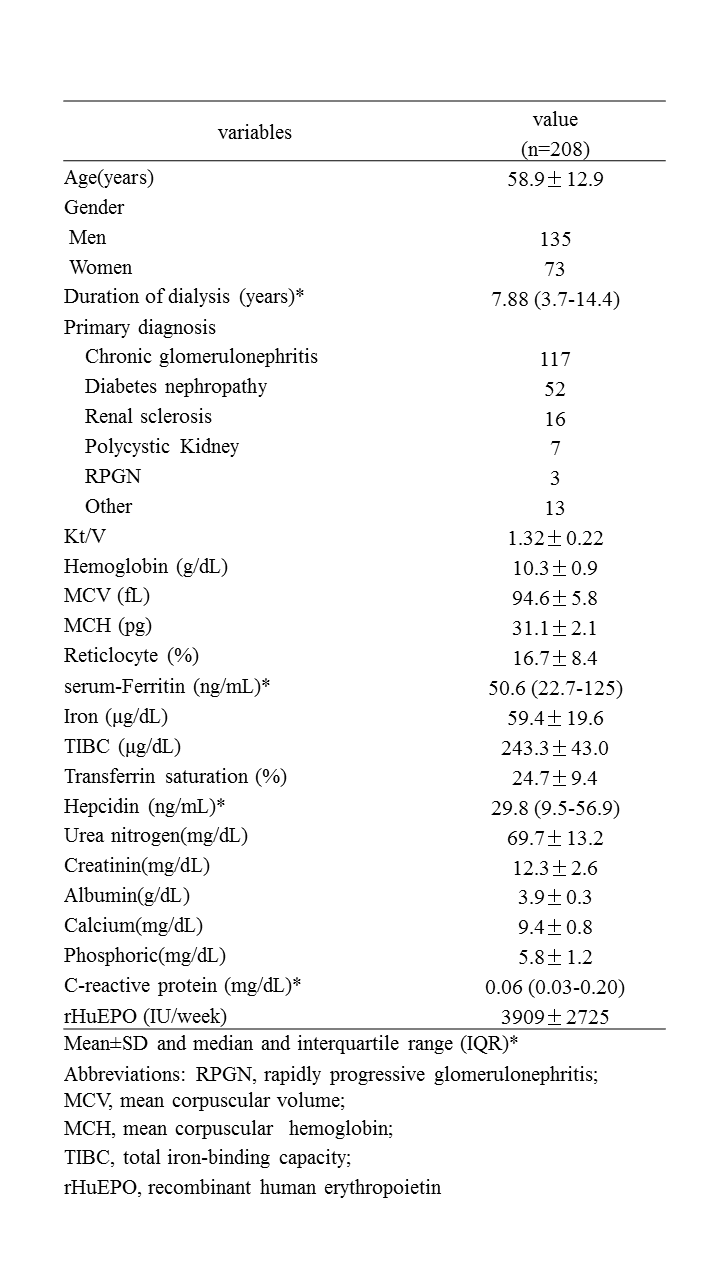

Supplement: S1 Table — (TIF) [file pone.0179608.s004.tif]

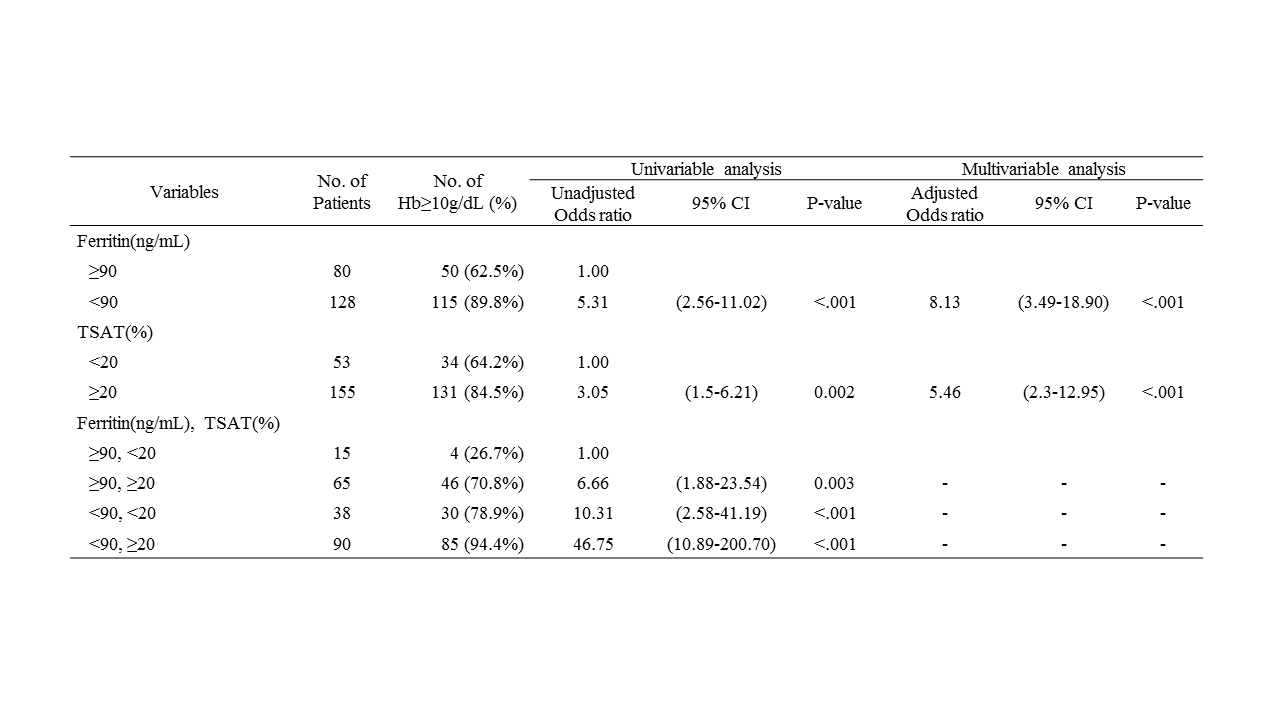

Supplement: S2 Table — (TIF) [file pone.0179608.s005.tif]

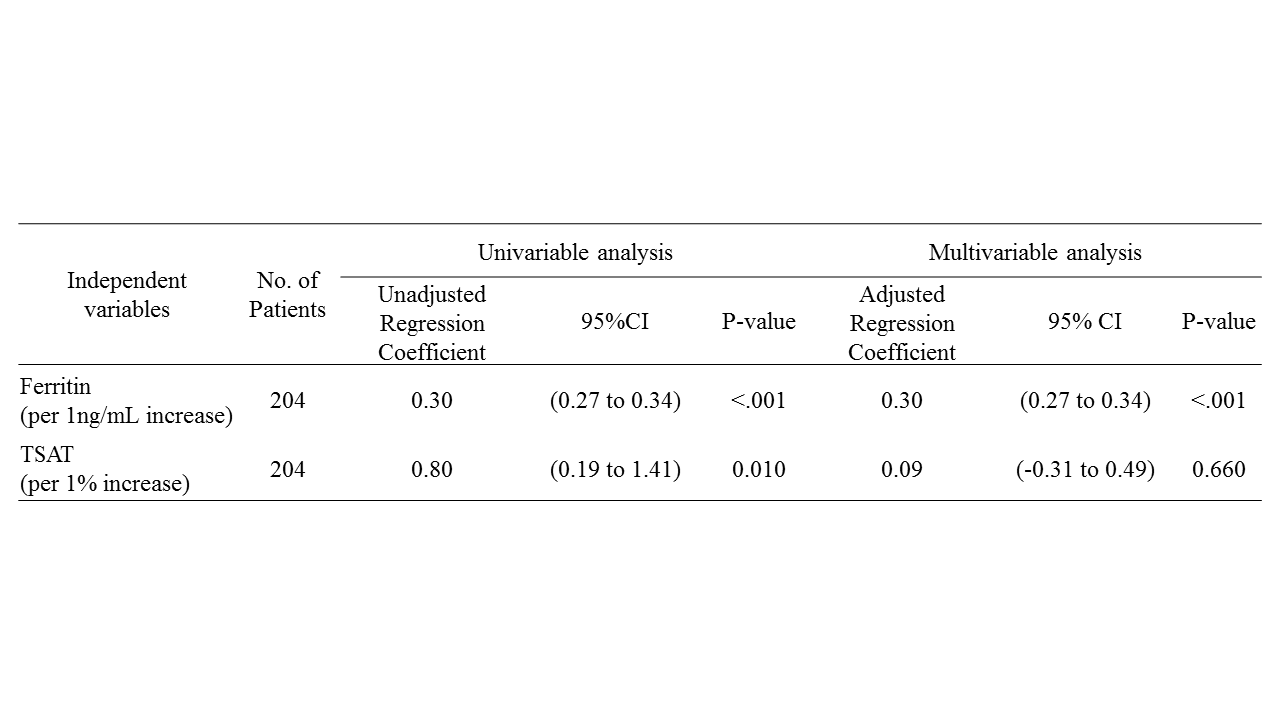

Supplement: S3 Table — (TIF) [file pone.0179608.s006.tif]
